# Tomato Residue Management from a Biorefinery Perspective and towards a Circular Economy

**DOI:** 10.3390/foods13121873

**Published:** 2024-06-14

**Authors:** Patrícia V. Almeida, Licínio M. Gando-Ferreira, Margarida J. Quina

**Affiliations:** CERES, Department of Chemical Engineering, University of Coimbra, 3030-790 Coimbra, Portugal; patriciav@eq.uc.pt (P.V.A.); lferreira@eq.uc.pt (L.M.G.-F.)

**Keywords:** resources recovery, circular economy, extraction, anaerobic digestion, composting, economic evaluation

## Abstract

The tomato industry is a relevant socio-economic activity in the European Union, while it generates a large variety of residues. Tomatoes unfit for consumption, tomato peels, seeds, industrial pomace, and plants are examples of residues of this industry. Commonly, some of the residues can be left in the field, composted, used for animal feeding, or valorized through anaerobic digestion. However, more economic value can be attributed to these residues if a biorefinery approach is applied. Indeed, many value-added compounds can be obtained by the integration of different processes while closing the carbon and nutrient loops. The extraction of bioactive compounds followed by anaerobic digestion and composting seems to be a viable proposal for a biorefinery approach. Thus, this study aims to review the biorefinery strategies for valorizing tomato residues, highlighting the main processes proposed. The recovery of lycopene, β-carotene, and phenolic compounds has been widely studied at the lab scale, while energy recovery has already been applied at the industrial scale. Although techno-economic analysis is scarce for tomato residue valorization processes, positive net present values (NPV) and low payback times (PBT) have been reported in the literature. Thus, more work comparing multiple extraction technologies and biorefinery strategies coupled with economic and environmental assessment should be performed to select the most promising management route for tomato residues.

## 1. Introduction

The increasing pressure from governmental agencies to avoid food waste and the environmental impact of agro-industrial residues have encouraged the scientific community to investigate different strategies for managing and valorizing those waste streams. Biorefinery has been highlighted as a relevant approach for this purpose [1,2]. The integration of well-established processes enhances the recovery of multiple value-added products, focusing on reuse, recycling, and valorization to recover energy. This contributes to the objectives defined in the European Circular Economy Action Plan (CEAP) and other frameworks, such as the European Green Deal (EGD).

The recovery of value-added compounds (proteins, pectin, antioxidants, carotenoids, phenolic compounds, etc.), energy, and nutrients from multiple agro-residues have been widely studied [3,4,5,6]. The use of processes such as extraction, fermentation, anaerobic digestion, pyrolysis, gasification, and composting are the main processes assessed to valorize agro-industrial residues [1,7,8,9,10,11]. However, only recently the integration of those processes has been assessed for some agro-industrial residues applying the biorefinery concept [12,13,14,15]. Although stand-alone processes have proved suitable to valorize the residues with high efficiencies, the use of sequential processes may improve or not the yields of the processes. Almeida et al. [12,13] proved that extracting phenolic compounds from tomato and wine residues did not impair the anaerobic digestion and pyrolysis performance. However, Ninčević Grassino [16] showed that extracting pectin before phenolic compounds drastically reduced the extracting yield. For this reason, the biorefinery viability must be done on a case-by-case basis including techno-economic analysis and life cycle assessment (LCA).

This study will focus on the case study of tomato industries that generate multiple residues including tomatoes unfit for consumption (rotten and green), peels, seeds, pomace, and tomato plants. Thus, the main objective is to summarize the outputs regarding the biorefineries of tomato residues present in the literature. In particular, updated findings will be highlighted regarding specific processes (extraction, anaerobic digestion, and composting) with the greatest potential to be used in biorefinery approaches. An overview of the tomato processing industries, the technological readiness of the processes, and the techno-economic analysis will also be assessed. The review of the tomato residues biorefineries and techno-economic analysis found in the literature for either stand-alone or integrated valorization processes are the main novelty of this work. To the best of the authors’ knowledge, there is no review paper highlighting the biorefinery approaches and economic analysis for tomato residue valorization.

## 2. Tomato Industry

**Figure 1:**
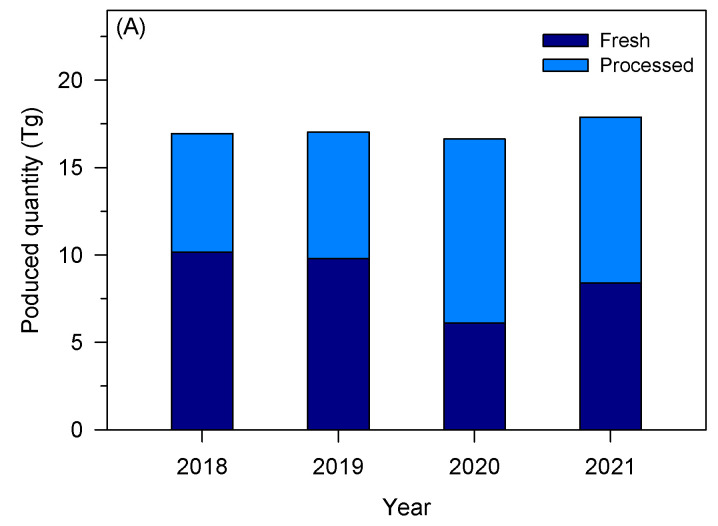

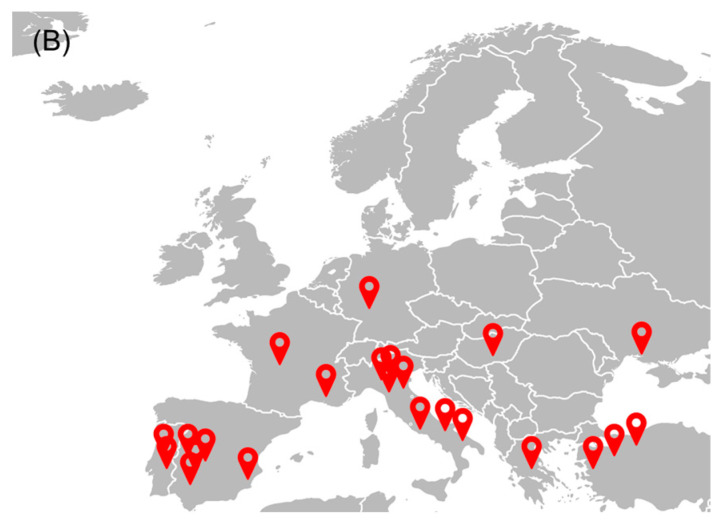
(**A**) Quantity of tomato produced to be consumed fresh and after processing in the EU (based on [17]); (**B**) main tomato processing industries in Europe within the top 50 companies in the sector worldwide (based on [20]).

The tomato industry is a relevant socio-economic activity in the European Union (EU), presenting a gross production value of about 91 billion euros [17]. After grapes, tomatoes are among the most produced vegetables and fruits in the EU, representing about 13% in 2022 [18]. Figure 1A shows the quantity of tomatoes produced and processed in the EU over four years. In 2021, about 16.9 Tg of tomatoes were harvested, while Italy, Spain, and Portugal are the top three producers of tomatoes, accounting for 40%, 29%, and 11% of the EU’s total production, respectively [17]. Although tomatoes can be consumed fresh, a large fraction (about 40%) is processed to obtain paste, sauce, puree, and ketchup, making the tomato processing industry also a relevant socio-economic activity. In 2022, the tomato processing industry presented a market value of around 40 billion euros in Western Europe, with an expected annual growth rate ranging between 0.4 and 1.8% [19]. Ready meals, followed by tomato paste, are the most tomato-derivative products produced in Europe [19]. The most relevant groups or companies in Europe are located mainly in Italy, Spain, and Portugal—Figure 1B. Sugal group, Conesa, and Mutti were the main tomato processing industries in Europe in 2020 [20].

**Figure 2:**
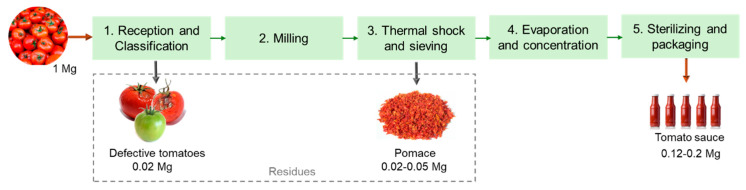
Schematic representation of a tomato processing industry (based on Lu et al. [22]).

The industrial process for tomato transformation into sauce or paste comprises multiple unit processes, as depicted in Figure 2. Globally, the tomatoes are first evaluated and selected, rejecting the defective tomatoes. Those that meet the quality requirements are driven with water through the production lines facilitating transport and the washing step. Then, the tomatoes are milled and subjected to a thermal shock to secure enzymatic inactivity, preventing product degradation. The peels and seeds are separated from the pulp through sieving with rotating blades. The pulp is then dehydrated using vacuum and temperature to obtain a concentrated product. In the final step, the products are subjected to high temperatures and quickly cooled for sterilization before being bottled [21]. Overall, these industries produce large amounts of residues in the sieving step, which is commonly named tomato pomace and is composed of peels, seeds, and some pulp. According to the literature, about 2–5% of the raw material weight corresponds to tomato pomace [22]. Thus, around 332 Gg of tomato pomace were generated by industries in the EU in 2021. During the selection and washing step, the immature, defective, or damaged tomatoes are also discarded, and the amount of rejection typically corresponds to 2% of the raw material weight [23].

Besides tomato pomace and defective tomatoes, during the harvest season, unripe tomatoes and tomato leaves and stalks are left in the agricultural field, as well as fully ripe tomatoes (rotten). About 15 Mg/ha∙year of residues, including unmarketable tomatoes, leaves, and stalks, may be produced in greenhouse systems [24]. Considering a harvested area of 208,630 ha [18], about 3.1 Tg of residues were generated in the EU during 2022. Moreover, tomatoes can also reach an advanced stage of maturation during commercialization, and all these streams are considered waste in the tomato industry [25].

## 3. Tomato Residue Composition

The main general physical and chemical characteristics of tomato residues are presented in Table 1. The five residues of tomato presented can be divided into two main categories: the fruit components (tomato rotten, unripe tomato, and tomato pomace) and the more lignocellulosic materials (tomato plant and tomato stalks).

The rotten tomato is mostly composed of water, with a moisture content ranging from 93.8–94.7%. However, 81.1–89.9% of the total solids remaining are organic matter, meaning that both materials have the potential to be biologically processed. The tomato fruit is acidic, with a pH of around 4.5, which must be considered when biological treatments are implemented due to its sensibility to pH. Tomato pomace (peels and seeds) has lower moisture levels (57.6–89.1%) but organic matter content (87.8–97.8%) in the same order as the rotten tomato. The structural carbohydrates (hemicellulose and cellulose), lignin, and protein contents of rotten tomatoes are similar to tomato pomace. According to Del Valle et al. [26], fiber, protein, and lipids are the main components of tomato pomace on a dry matter basis.

Tomato plants and tomato stalks have a similar composition because the whole plant includes the stems. The natural pH of both residues rounds the neutrality (5.4–7.6), and moisture content is between 80.3–92.4%. Similar to tomato fruit and tomato pomace, the plant is rich in lignin (7.6–28.5%), hemicellulose (6.4–16.9%), cellulose (15.5–33.1%), and proteins (13.8–18.6%).

**Table 1:** General characterization of tomato rotten, pomace, plant, and stalks.

Parameter	Tomato Rotten ^a^	Unripe Tomato ^b^	Tomato Pomace ^c^	Tomato Plant ^d^	Tomato Stalks ^e^
pH	4.4–4.6	4.0	4.6–4.7	5.4–7.6	7.9
Moisture (%)	93.8–94.7	92.2	57.6–89.1	80.3–92.4	20.77–80.3
Organic matter (%, db)	81.1–89.9	88.0	87.8–97.8	75.9–79.8	63.6–86.3
Ash (%, db)	15.8–18.9	-	3.2–4.1	16.0–20.2	13.7
Lignin (%, db)	2.43–19.9	4.11	13.5–37.3	7.6–28.5	11.9–17.9
Hemicellulose (%, db)	3.4–15.2	31.02	5.4–14.4	6.4–16.9	7.9–20.4
Cellulose (%, db)	8.6–16.2	23.15	7.7–34.0	15.5–33.1	26.5–43.1
Lipids (%, db)	2.3–3.4	2.75	5.0–11.0	2.2–11.9	-
Proteins (%, db)	17.4	17.35	16.6–30.0	13.8–18.6	-
Pectin (%, db)	-	-	7.5–8.8	-	-
C (%,db)	42–44	38	57	-	37
N (%, db)	2.4–3.9	2.0	0.92	-	1.6–2.8
H (%, db)	5.7–6.5	6.0	7.5	-	5.1
O (%, db)	27–35	42	35	-	35
Phenolic compounds					
Caffeic acid (mg/g_db_)	-	-	0.051–1.82	0.14–1.5	0.25–1.2
p-coumaric acid (mg/g_db_)	-	-	0.0114–2.33	-	-
Chlorogenic acid (mg/g_db_)	-	-	0.04–0.52	0.25–3	0.25–1.1
Ferulic acid (mg/g_db_)	-	-	0.0023–0.45	-	-
Gallic acid (mg/g_db_)	-	-	-	0.76–4.636	0.75–1.0
Naringenin (mg/g_db_)	0.029–25.22	-	-	-	-
Quercetin (mg/g_db_)	0.0373–5.89	-	-	-	-
Rutin (mg/g_db_)	0.0292–1.255	-	-	0.15–3.02	0.2–1.3
Catechin (mg/g_db_)	0.18–1.23	-		-	-

^a^ References [5,12,13,27,28,29,30,31,32,33,34,35,36,37,38,39]; ^b^ [5,12,13]; ^c^ [26,33,34,35,36,40,41,42,43,44,45,46,47,48,49,50]; ^d^ [51,52,53,54,55,56]; ^e^ [56,57,58,59,60,61,62,63].

In smaller quantities but with higher value, tomato residues contain carotenoids (β-carotene, lycopene, and lutein) and phenolic compounds. Carotenoids are organic pigments produced by plants and microorganisms and can be divided into two major groups: carotenes and xanthophylls. They are responsible for the yellow, orange, and red colors of many fruits and vegetables. The carotenoid content in tomatoes is affected by agronomic, environmental factors, variety, and ripening stage [64,65]. In addition, the diverse parts of the tomato (peel, pulp, and seeds) can present substantial differences in carotenoid concentration [64], and the extraction process applied significantly affects the result. Those are the main reasons to find a large variability of carotenoid content reported in different studies. This topic will be discussed in depth in Section 4.1.

Also, the phenolic compounds in tomato residues are dependent on the variety of the fruit, the agronomic conditions (light, irrigation, temperature), the maturation stage, the extraction process, and respective conditions (solvent, time), pre-treatments applied, and analytical methodology [12,36,65]. This dependency can explain the wide range of phenolic contents reported. Due to this variability, it is difficult to conclude if tomato residues are richer in flavonoids than phenolic acids or the contrary. Indeed, different authors have reported distinct results—Table 1 [33,36,64,66,67]. Although the varying factors hinder the comparison, an increase in phenolic concentration from tomato seed to tomato peel can be noted [36,67]. Among the phenolic acids, caffeic acid, p-coumaric acid, chlorogenic acid, and ferulic acid are the molecules found in concentrations of potential interest for recovering from tomato pomace [34,35,50,68]. Gallic acid and vanillic acid are also found in tomato residues but in minor amounts. Naringenin, quercetin, rutin, and catechin are the most abundant flavonoids present in tomato fruit residues [32,33,34,35,37,38,39,68]. Catalkaya and Kahveci [50] reported a wide range of rutin concentrations (5–88.1 mg/g_dbe_—dry basis extract) according to the solvent used, proving the influence of extraction methodology on the phenolic compound profiler.

Phenolic extraction from tomato plants (leaves and stems) has been scarcely studied. Silva-Beltrán et al. [56] analyzed the phenolic profile of different parts of two tomato plants, namely leaves, stems, and their mixture. Gallic acid, chlorogenic acid, caffeic acid, and rutin are the phenolic compounds identified in the tomato plant, in concentrations between 0.76–4.636, 0.25–3, 0.14–1.5, and 0.15–3.02 mg/g_db_, respectively. Quercetin and ferulic acid were also identified but in lower concentrations. Considering this study, tomato leaves present the highest phenolic content in the tomato plant.

An increasing demand for these bioactive compounds has been noticed because of the growing awareness of a healthy lifestyle. Indeed, carotenoids and phenolic compounds present biological properties of high interest for the food, cosmetic, and pharmaceutical industries. Because carotenoids are natural pigments, they are used as colorants in foods and cosmetic products [69]. Carotenoids also play an important role in the production of nutraceuticals such as vitamin A (responsible for the protection of the retina from blue and near-ultraviolet light) [69]. It presents high antioxidant activity, enabling its use as a preservative, and some studies revealed that diets rich in carotenoids reduce the risk of prostate cancer [70], benefit osteoporosis [71], and prevent cardiovascular diseases [71]. Phenolic compounds showed antioxidant, anti-inflammatory, and anti-cancer properties [1].

## 4. Biorefinery Approach for Tomato Residue Valorization

**Figure 3:**
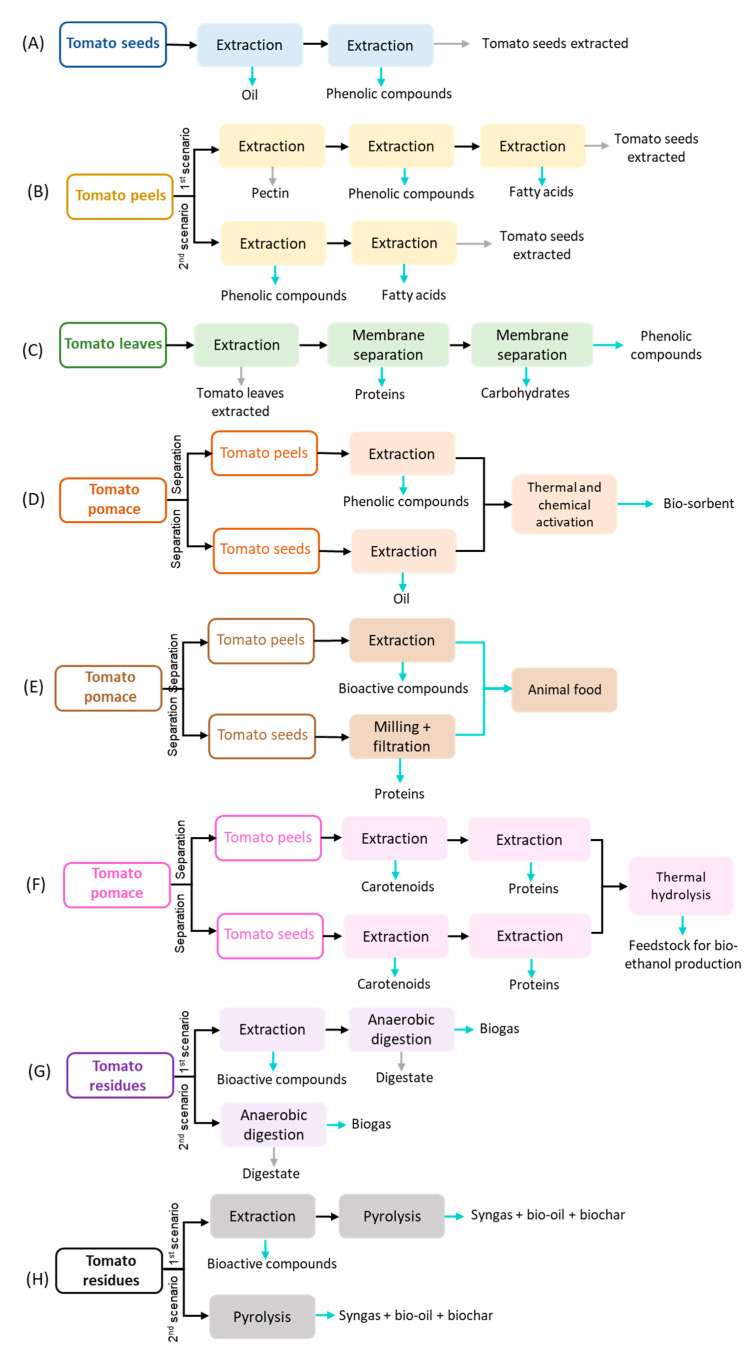
Biorefinery approach proposed to manage and create value for tomato residues based on Ouatmani et al. [82] (**A**); Ninčević Grassino [83] (**B**); Bazzarelli et al. [84] (**C**); Azabou et al. [85] (**D**); Baker et al. [49] (**E**); Kehili et al. [86] (**F**); Almeida et al. [12]; Allison and Simmons [87] (**G**); and Almeida et al. [13] (**H**).

Currently, tomatoes are unfit for consumption, and tomato plants are left in the agriculture field after crop cultivation [12], while at the industrial level, tomato pomace is commonly used for animal feed [72]. More recently, tomato residues have also been used as a substrate in anaerobic digestion to recover energy [73]. Indeed, presently, there are many alternative processes to valorize these residues: extraction of value-added compounds (e.g., pectin, oil, lycopene, β-carotenes, phenolic compounds…), pyrolysis, gasification, anaerobic digestion, and composting. Table 2 summarizes the technology readiness (TRL) of the main technologies used for treating/valorizing tomato residues based on the literature. Many patents were found aiming to extract carotenoids (including lycopene) from tomato residues, considering different strategies. Multiple pre-treatments (WO2003020053A1, US20140316175A1), solvent mixtures (US20100055261A1), and non-conventional extraction processes (CN1799674, CN1334328A, CN101298618A, and CN101121631A) have been claimed in those patents. Proof of their industrial application was not found in the literature. Indeed, 90% of carotenoids are chemically synthesized (US20210115454A1). Although all the technologies mentioned in Table 2 are well-established and may be industrially used for managing tomato residues, valorization may not be maximized with a single process. As mentioned before, the integration of multiple processes aiming at a biorefinery approach may enhance the valorization of tomato residues. Some authors have already studied different biorefinery strategies using tomato residues, and Figure 3 summarizes eight possible approaches.

All the biorefineries proposed in the literature using tomato residues include the extraction of valuable compounds, such as carotenoids, phenolics, oil, pectin, and proteins. Ouatmani et al. [82] and Ninčević Grassino et al. [83] proposed a multistage extraction to recover different value-added compounds (oil, phenolic compounds, pectin, and fatty acids). Ouatmani et al. [82] evaluated the extraction of oil (with a yield of 21.69%) from tomato seeds followed by a microwave extraction to recover phenolic and antioxidant compounds (2.31 mg GAE/g_db_; 78% of antioxidant activity). Ninčević Grassino [83] studied the effect of removing pectin from tomato peels on phenolic compounds and fatty acids recovery. Indeed, it was proven that tomato peels can be used to extract sequentially pectin (21.7%), phenolic compounds (40.64 mg GAE/g_db_), and fatty acids (31.67%, including palmitic acid and linoleic acid). On the contrary, the use of high hydrostatic pressure extraction for pectin recovery counters this conclusion. The recovery efficiency of phenolic compounds from tomato pomace primarily extracted with high hydrostatic pressure for pectin removal was lower than tomato pomace without other pre-processing. The high hydrostatic pressure extraction for tomato pomace affected the recovery yield of phenolic compounds probably because the interest molecules were extracted along with pectin, degraded, or formed complexes with pectin at high pressures [16]. A different strategy for obtaining multiple products was proposed by Bazzarelli et al. [84] The integration of the extraction process with two consecutive membrane systems was used to separate proteins, carbohydrates, and phenolic compounds from tomato leaves. Azabou et al. [85], Baker et al. [49], and Kehili et al. [86] evaluated the valorization of tomato pomace by processing peels and seeds separately. Azabou et al. [85] extracted tomato peels and seeds to obtain bioactive compounds (phenolic compounds: 199.35 mg GAE/g_dbe_ and lycopene: 0.0367 mg/g_db_) and oil (17.15%), respectively. The remaining tomato peel and seed extracted were mixed and subjected to a thermal treatment to produce a biosorbent for dye removal. A zero-waste path was evaluated in this work. Although Baker et al. [49] do not assess the biorefinery proposed, a zero-waste path is also presented. Briefly, the tomato peels are extracted to obtain carotenoids, while tomato seeds are treated with grinders and membrane processes to recover proteins. The remaining solids (from tomato seed and peel) are mainly composed of fibers that can be used for animal feed. The sequential extraction of carotenoids and proteins was evaluated by Kehili et al. [86] Indeed, the purity of proteins recovered was higher after the supercritical fluid extraction. The solid spent of the extraction was submitted to a thermal treatment to hydrolyze the cellulose and hemicellulose into smaller molecules that can be used in fermentation processes to produce bioethanol. Kehili et al. [86] concluded that raw tomato seeds and peels lead to higher concentrations of sugars than extracted solids. Even so, an extensive techno-economic analysis should be performed to realize which strategy would be the most profitable. Allison and Simmons [87] and Almeida et al. [12,13] conjugated the extraction process with an energetic valorization process for three different types of tomato residues. The studies concluded that the extraction of bioactive compounds (phenolic compounds, β-carotene, and lycopene) did not negatively affect the energetic recovery compared to a stand-alone process. Thus, bioactive compounds can be recovered without compromising the energetic value either through anaerobic digestion or pyrolysis.

**Figure 4:**
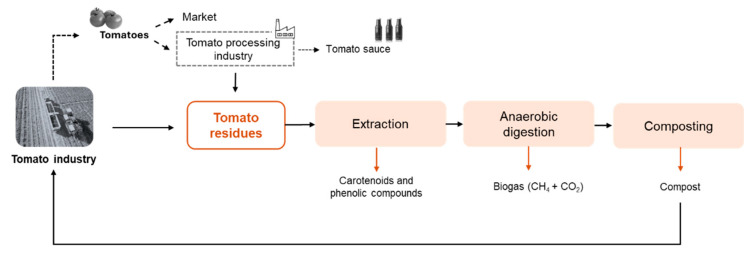
Biorefinery approach proposed in the present study to manage and create value for tomato residues (tomato rotten, green, peels, seeds, pomace, and tomato plant).

These technological configurations are far from being industrially applied. The comparison between them is hard due to the diverse combinations and results obtained. Moreover, an economic analysis coupled with an LCA should be done to support the selection of the best biorefinery proposition. Even so, this is a recent topic that needs to be investigated in depth. More studies and alternatives can be explored first, aiming to maximize the routes of valorization. The authors of the present study propose a strategy to manage and create value from tomato residues (Figure 4), which allows the carbon cycle (and other nutrients) to be closed. The proposal includes the extraction of bioactive compounds (phenolic compounds, β-carotenes, and lycopene), followed by anaerobic digestion, producing biogas, and composting process to stabilize the organic matter of digestate, generating a stable compost. In addition, many other processes can be added to the biorefinery approach to separate and purify the products. For example, the extract rich in bioactive compounds can be treated by sequential membrane processes to divide the main compounds into single products, as Bazzarelli et al. [84] proposed. The fractionation of extracts obtained from tomato residues has been scarcely studied through membrane processes [84]. However, microfiltration, ultrafiltration, and nanofiltration have been used to separate proteins, carbohydrates, and phenolic compounds from agro-food by-products (olive-mill wastewater, orange press liquor, grape pomace, and others) [88]. Although the separation and purification of value-added compounds is possible, the crude extract presents a mixture of bioactive compounds that may present positive synergistic effects, enhancing the beneficial properties of the molecules. Moreover, separating and purifying increases the operating costs; thus, the application of the crude extract should be prioritized whenever possible [1].

### 4.1. Recovering Value-Added Compounds through Extraction Processes

Solid-liquid extraction processes (SLE) are commonly used to recover carotenoids and phenolic compounds from tomato residues. These processes separate the soluble molecules present in the solid matrix through solvent contact. The molecules of interest are dissolved in the solvent, while diffusion mechanisms occur until equilibrium is reached. All strategies that improve the solubility of the molecule and diffusion of the solvent enhance extraction efficiency [89]. The operating conditions of SLE for recovering lycopene and β-carotene from tomato residues are summarized in Table 3. Hexane, acetone, methanol, and ethyl lactate are the most used solvents for extracting carotenoids. Mixtures with some of those solvents have enhanced the extraction efficiencies. Indeed, a mixture of 50% hexane and 50% ethyl acetate allowed an extraction efficiency of 16% and 30% higher than individual ethyl acetate and hexane, respectively [90]. A similar trend was observed for other solvents [90].

In addition to the solvent concentration, other operating conditions play an important role in extraction efficiency. Typically, higher temperatures, liquid-solid (L/S) ratios, and time of contact lead to high extraction yields [91]. Even so, room temperature and L/S ratios between 10 and 100 mL/g have been commonly used among conventional extraction processes to recover carotenoids.

Non-conventional extraction processes, such as ultrasound-assisted extraction (UAE), microwave-assisted extraction (MAE), and supercritical fluid extraction (SFE), have also been studied to recover carotenoids from tomato residues. Design of experiments (DoEs) is one of the most used methodologies to optimize the operating conditions for maximizing the recovery of carotenoids [91,92,93,94]. More recently, the DoE was also applied to compare different SLE processes aiming to select the most suitable [93].

According to Kumcuoglu et al. [91], time is the most important factor in UAE. The lycopene yield increased sharply in the first minutes, whereas after 20 min, the yield remained constant [91]. UAE allows for obtaining similar carotenoid yields while reducing the extraction time by 1.5 to 3.0 times compared with the conventional extraction method [95]. Large sonication times (>40 min) can cause sample deterioration [96].

MAE can also reduce the time of extraction and enhance the lycopene yield. Indeed, Ho et al. [97] obtained about 170 mg/kg_db_ of lycopene in 1 min with MAE, while 138 mg/kg_db_ was achieved in 30 min with the conventional process.

SFE was studied as an alternative to extraction processes based on toxic organic solvents. Multiple units have been used to report the results obtained through SFE, hindering its comparison (Table 3). Nevertheless, Popescu et al. [98] obtained extracts richer in lycopene content through conventional extraction (4500 mg/kg_dbe_) than SFE (2500 mg/kg_dbe_). Ubeyitogullari and Ciftci [99] reported statistically similar carotenoid content extracted through SFE and hexanic extraction. However, the bioaccessibility of lycopene in the extracts was enhanced through SFE compared with hexanic extraction.

Other strategies have been evaluated to improve the extraction of carotenoids, including sequential extractions and pre-treatments. Strati and Oreopoulou [100] revealed that in the second and third extraction step about 20–30% and 5–15%, respectively, of the total carotenoid is still recovered. Drying and milling are the most common pre-treatments applied. Indeed, most of the authors dry and mill the tomato samples before studying the extraction. Nonetheless, Allison and Simmons [87], Chada et al. [93], and Lazzarini et al. [101] evaluated the effect of different drying methods. According to Allison and Simmons [87], higher carotenoid concentrations were obtained for vacuum-dried pomace (293–476 mg/kg_db_). For solar-dried pomace, the lycopene content was below the detection limit (<5 mg/kg_db_). Freeze-drying and non-thermal air drying enhanced (about 2.38–6.08 times) the recovery of lycopene compared to non-drying and heat drying (85 °C, 2 h) [101]. Moreover, non-drying revealed higher concentrations (34.11 mg/kg_dbe_) of lycopene than heat drying (15.87 mg/kg_dbe_). The selection of the drying process, as well as the temperature, is relevant because the carotenoids are sensitive to high temperatures. Authors have shown that temperatures higher than 70 °C increase the degradation rate of lycopene and β-carotene [102,103,104,105]. Thus, oven drying under 70 °C is highly recommended.

**Figure 5:**
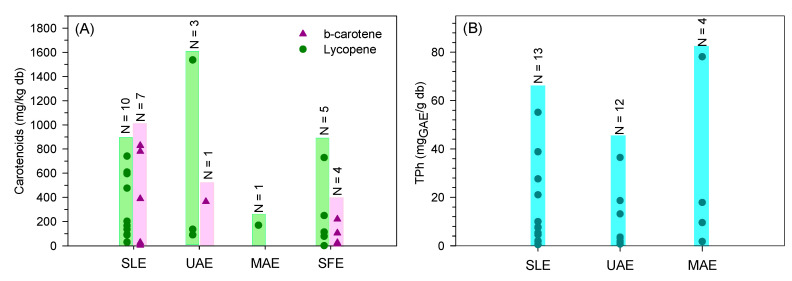
Distribution of (**A**)—carotenoids and total phenolic content, (**B**)—obtained through conventional extraction (SLE), ultrasound-assisted extraction (UAE), microwave-assisted extraction (MAE), and supercritical fluid extraction (SFE) found in the literature (N represents the number of results from the literature).

The wide range of β-carotene and lycopene content extracted from tomato fruit residues is well visible in Figure 5A. Indeed, the different operating conditions and units used in the studies are responsible for the variability of results. Furthermore, the lycopene and β-carotene content strongly depend on the tomato variety. Adalid et al. [106] studied the composition of 49 varieties of tomatoes and reported a large variability in lycopene and β-carotene content ranging between 0.4–167 and 2.9–14.6 mg/kg_db_, respectively. Even so, no clear evidence is noted among the different solid-liquid extractions (conventional, UAE, MAE, and SFE). A more concise and systematic study comparing the multiple technologies and an economic evaluation should be done to select the most promising extraction process to recover carotenoids.

**Table 3:** β-carotene and lycopene content (mg/kg_db_) achieved by SLE of tomato residues.

Ref.	Operation Conditions	Residue	β-Carotene	Lycopene	Total Carotenoids
*Conventional extraction*					
[87]	Solvent: hexane:acetone:ethanol (50:25:25%); T: 100 °C; t: 90 min; L/S: 100; d_p_: <1 mm	Pomace	-	476	-
[90]	Solvent: hexane:ethyl acetate (50:50%); T: 25 °C; t: 30 min; L/S: 10; d_p_: <1 mm	Pomace	4.75	30.3	36.5
[91]	Solvent: hexane:methanol:acetone (50:25:25%); T: 20–60 °C; t: 10–40 min; L/S: 20–50; d_p_: 0.286 mm	Pomace	-	93.9	-
[100]	Solvent: ethyl lactate; T: 25 °C; t: 40 min; L/S: 10; d_p_: <1 mm	Pomace	-	203	-
[107]	Solvent: hexane; t: 12 h; L/S: 30; d_p_: <0.3 mm	Peels	27.9	609	-
[95]	Solvent: acetone; T: 20 °C; t: 20 min; L/S: 30; d_p_: <1 mm	Peels	389	136	568
[98]	Solvent: ethyl acetate; L/S: 12.5	Tomato	~9500 ^b^/828	~8500 ^b^/741	-
		Pomace	~5900 ^b^/780	~4500 ^b^/595	-
[106]	Solvent: ethanol: hexane (57:43%); t: 60 min; L/S: 70; d_p_: <0.4 mm	Tomato	14.6	167	-
[108]	Solvent: Capric acid/lauric acid (33:67%); t: 62 min; L/S: 61	Tomato	-	79.9 ^a^	-
[108]	Solvent: acetone; L/S: 61	Tomato	3.7 ^a/^4.0	81.5 ^a^/89	88.6 ^a^/96.3
[12]	Solvent: hexane; t: 6 h; L/S: 67	Rotten	53.2 ^b^	6.08 ^b^	-
		Green	8.6 ^b^	2.57 ^b^	-
		Plant	53.2 ^b^	13.8 ^b^	-
[109]	Solvent: ethanol:acetic acid (95:5%); T: 25 °C; t: 72 h	Stem	-		1772 ^b^
		Leaves	-		5354 ^b^
		Root	-		493 ^b^
		Plant	-		2845 ^b^
*Ultrassoud-assisted extraction*					
[91]	Solvent: hexane:methanol:acetone (50:25:25%); t: 1–30 min; L/S: 20–50; power: 50–90 W; d_p_: 0.286 mm	Pomace	-	90.1	-
[92]	Solvent: ethanol; t: 20 min; T: 65 °C; L/S: 72; amplitude: 65; d_p_: <0.732 mm	Pomace	-	1536	1408
[95]	Solvent: acetone; T: 20 °C; t: 5–20 min; L/S: 30; Power: 45 kHz; d_p_: <1 mm	Peels	365	136	539
[101]	Solvent: acetone:n-hexane; t: 20 min; T: 63 °C; S/L: 0.1	Pomace	5717.46 ^b^	96.55 ^b^	-
[96]	Solvent: acetonitrile:methanol (80:20%); T: 20–30 °C; t: 45 min; L/S: 180; power: 100 W	Green	30 ^a^	45 ^a^	-
		Red-ripe	80 ^a^	700 ^a^	-
*Microwave-assisted extraction*					
[97]	Solvent: ethyl acetate; t: 1 min; L/S: 20; power: 400 W; energy: 24 kJ; d_p_: <5 mm	Pomace	-	170	-
*Supercritical fluid extraction*					
[98]	Solvent: CO_2_; P: 400 bar; T: 70 °C; solvent flow rate: 11 kg/h; mass sample: 180 g	Tomato	~2000 ^b^/105	~1500 ^b^/79	-
		Pomace	~2200 ^b^/220	~2500 ^b^/250	-
[99]	Solvent: CO_2_; T: 80 °C; t: 20 min; mass sample: 12 g; P: 300 bar; solvent flow rate: 1 L/min	Pomace	220 ^b/^25.3	990 ^b^/114	-
[110]	Solvent: CO_2_; T: 80 °C; t: 20 min; P: 500 bar; solvent flow rate: 30.4 mg/s; d_p_: <1 mm	Pomace	-	1.32 ^a/^1.4	
[107]	Solvent: CO_2_; t: 180 min; mass sample: 10 g; P: 400 bar; solvent flow rate: 4 g/min; d_p_: <0.3 mm	Peels	16.4	729	-
*Enzymatic-assisted extraction*					
[94]	Enzyme: 1.4%; time: 92 min; T: 52 °C	Pomace	-	3996	-

^a^ mg/kg_fw_—fresh weight; ^b^ mg/kg_dbe_—dry basis extract; S/L—solid-liquid ratio in mL/g; d_p_—avarage particle diameter.

The recovery of phenolic compounds from tomato residues through SLE and non-conventional SLE processes are summarized in Table 4 and Table 5, respectively. Similar to the carotenoids, many authors have also used DoEs to evaluate the operating conditions that maximize the extraction of phenolic compounds [50,111,112]. To synthesize the information, only the best results are presented in Table 4 and Table 5.

Total phenolic content (TPh) has been reported in a range of 0.72–78.06 mg_GAE_/g_db_ for tomato peel, 0.67–2.03 mg_GAE_/g_db_ for seed, 1.72–9.55 mg_GAE_/g_db_ for whole fruit and 0.43–55.1 mg_GAE_/g_db_ for tomato pomace. A reduced number of studies evaluating the phenolic extraction from tomato plants was found. Nevertheless, 1.71 mg_GAE_/g_db_ was reported [109]. Arab et al. [113] measured higher contents of phenolics in tomato leaves (162–240 mg_GAE_/g_db_).

Water, ethanol, methanol, and acetone are the main solvents used for phenolic compound extraction. The diffusion of phenolics from the solid to the liquid matrix is promoted by diluted organic solvents [16,114]. Indeed, total phenolic content recovery increased from 14.56 to 36.44 mg_GAE_/g_db_ when the solvent used was 96% and 70% ethanol, respectively [16].

Besides the solvent, temperature and time are also important operational conditions because, typically, high extraction times and temperatures enhance the traditional SLE efficiency. Ninčević Grassino et al. [83] evaluated the influence of time and revealed an increase in the total phenolic and total flavonoid content from 27.62 to 38.79 mg_GAE_/g_db_ and 2.21 to 4.80 mg_QE_/g_db_ when time rises from 3 to 12 h, respectively. Temperatures between 20 and 60 °C are commonly applied to extract phenolic compounds from tomato residues. Also, the L/S ratio, as reported before, is an important factor affecting the extraction yield, and 5–60 mL/g is the range commonly reported.

**Table 4:** Total phenolic compounds (TPh—mg_GAE_/g_db_) and total flavonoid content (TFC—mg_QE_/g_db_) achieved by a solid-liquid extraction of tomato residues.

Ref.	Operation Conditions	Residue	TPh	TFC
[50]	Solvent: ethyl acetate:ethanol (50:50) t: 30 min; T: 25 °C L/S: 5	Pomace	27.6 ^b^	
[115]	Solvent: methanol (95%); t: 30 min; T: 50 °C; L/S: 50	Pomace	5.3	2.21
[116]	Solvent: ethanol:water (99.5:0.05%) t: 6 h; T: 25 °C; L/S: 60	Pomace	55.1/199 ^b^	102 ^b^
[117]	Solvent: methanol:water (80:20%) t: overnight; T: 25 °C; L/S: 10; d_p_: <0.5 mm	Pomace	0.43	-
	Solvent: ethanol:water (50:50%); t: 20 min; T: 20 °C; L/S: 10	Pomace	0.028 ^a^	-
[118]	Solvent: ethanol:water (50:50%); t: 20 min; T: 20 °C; L/S: 10Pre-treated with a pulsed electric field (200 Pulses, 2.0 kV/cm)	Pomace		
			0.056 ^a^	-
[83]	Solvent: ethanol:water (70:30%) t: 12 h	Peel	38.79	4.80
[119]	Solvent: methanol (80%); T: 60 °C	Peel	21	-
[120]	Solvent: ethanol:water (70:30%)	Seed	2.03	-
[35]	Solvent: ethanol (80%); t: 1 h; T: 25 °C; L/S: 5	Tomato	115 ^b^/7.7	-
		Pomace	104 ^b^/9.96	-
[121]	Solvent: ethanol:water (70:30); t: 24 h; T: 25 °CPre-treated with microwaves during 30 sPre-treated with microwaves during 300 s	Tomato	1.92 ^a^	2.34 ^a^
			1.97 ^a^	2.58 ^a^
			6.95 ^a^	10.68 ^a^
[122]	Solvent: acetone; t: 1 h; T: 60 °C; L/S: 20	Tomato	0.278 ^a^	-
[123]	Solvent: water; t: 2 h; T: 90 °C	Tomato	7.56 ^b^	
[12]	Solvent: ethanol; t: 4 h; T: 50 °C; L/S: 20	Tomato	27.5 ^b^/4.5	-
		Green	9.90 ^b^/1.72	-
		Plant	27.1 ^b^/1.71	-
[27]	Solvent: water; t: 6 h; T: 0 °C; L/S: 20; d_p_: <1 mm	Rotten	14.2 ^b^/7.5	-
[124]	Solvent: methanol; t: 24 h; L/S: 5	Green	0.225 ^a^	0.184 ^a^
		Tomato	0.205 ^a^	0.210 ^a^
[109]	Solvent: ethanol:acetic acid (95:5%); t: 72 h; T: 25 °C	Stem	45 ^b^	28 ^b^
		Leaves	125.5 ^b^	61.96 ^b^
		Root	24.5 ^b^	19 ^b^
		Plant	75 ^b^	52 ^b^

^a^ Phenolic concentration in mg/g_fw_; ^b^ phenolic concentration in mg/g_dbe_; L/S—liquid-solid ratio in mL/g; d_p_—avarage particle diameter.

UAE and MAE have been widely studied as an alternative to traditional SLE. Ethanol concentration and time of sonication were considered the most important factors in UAE [16,112]. A non-linear relationship between total phenolic compounds and ethanol concentration was reported by Solaberrieta et al. [112] Ethanol concentrations of around 60–70% improved the extraction of phenolic compounds [16,112]. Time of sonication between 5 to 15 min was studied [16,112] and a linear correlation with phenolics was reported. A rise of about 16% in TPh was noted when the time of sonication increased from 5 to 15 min. Longer extraction times (about 1 h) in UAE were also used in other works [34,66], but a wide range of results for tomato pomace was reported probably because other operating conditions and the tomato variety are different.

Bakić et al. [114] used MAE with a power range of 0 to 500 W to maintain the temperature in the defined setpoint (25, 55, or 90 °C) and short periods (5 and 10 min). Higher extraction temperatures lead to richer extracts in phenolic and flavonoid content. A rise of 55 °C to 90 °C induced an increase in bioactive concentration from 55.44 to 78.06 mg_GAE_/g_db_ [114]. Using the DoE methodology, Solaberrieta et al. [112] evaluated and optimized time, temperature, ethanol concentration, and L/S ratio in the MAE of total phenolic content. Ethanol concentration and temperature were the most important operating conditions. Diluted ethanol (63%) and high temperatures (80 °C) maximized the TPh (1.72 mg/g_db_) obtained through MAE from seeds [112]. Although for different tomato residues, Solaberrieta et al. [112] and Plaza et al. [111] compared the optimization of UAE with MAE and both reported similar phenolic compounds recovery. Besides operating conditions, the ripening stage and pre-treatments also explain the wide range of results reported (Figure 5B).

The variety of tomato fruit and tomato plants led to different phenolic recoveries even when the same extraction conditions were used [37,67,109,113,125]. For example, the total phenolic content for different species of tomato leaves ranged from 83.35–125.5 mg_GAE_/g_dbe_ [109].

Another important aspect to be highlighted is the fact that the total phenolic content does not follow a well-defined trend regarding the tomato maturation stages [65,124]. Among the four stages (green, green-orange, orange-red, and red-ripe), the total phenolic content from the HLY 18 tomato variety increases from green to orange-red and then decreases until the red-ripe stage [124]. In contrast, for the Lyco 2 tomato, the total phenolic concentration remains constant during the ripening stages, a pattern also reported by Helyes and Lugasi [126]. As the same recovery process was used, the tomato variety determines the phenolic evolution in the different ripening stages. Raffo et al. [127] reported a decrease in phenolic concentration during the maturation process. Overall, no conclusions can be drawn about phenolic evolution during the ripening process.

Drying is the pre-treatment common to all the extraction studies of TPh from tomato residues. Similar to the recovery of carotenoids, the yield of phenolics extraction is also affected by the drying process. Segoviano-León et al. [66] reported a TPh of 242.3 mg_GAE_/g_db_ for extracting oven-dried tomato pomace, while freeze-dried tomato pomace reached a TPh of 508.3 mg_GAE_/g_db_.

In summary, the wide range of values reported (Figure 5B) shows that similar results can be achieved by any of the processes (conventional—SLE, UAE, and MAE). More concise studies optimizing the operating conditions while comparing the different extraction processes should be done to select the most promising one at an industrial scale. Moreover, an economic analysis should be performed, including the extraction of tomato residues without pre-drying.

**Table 5:** Total phenolic compounds (TPh—mg_GAE_/g_db_) and total flavonoid content (TFC—mg_QE_/g_db_) achieved by an ultrasound-assisted extraction (UAE), microwave-assisted extraction (MAE), accelerated solvent extraction (ASE), and enzymatic-assisted extraction (EAE) of tomato residues.

Ref.	Operation Conditions	Tomato	TPh	TFC
*Ultrasound-assisted extraction (UAE)*				
[16]	Solvent: ethanol: water:(70:30); t: 15 min; L/S: 50	Pomace	36.44	3.58
[33]	Solvent: methanol; L/S: 10;	Pomace	3.2	-
[34]	Solvent: methanol; t: 50 min; T: 25 °C L/S: 16.7; d_p_: <1 mm	Pomace	1.23	0.42
[111]	Solvent: water; t: 60 min; T: 25 °C; L/S: 10; amplitude: 50%	Pomace	18.65	-
[128]	Solvent: n-hexane; t: 2 min; T: 25; re-extracted with ethanol during 24 h	Pomace	13.15	-
[67]	Solvent: methanol; t: 1 h; L/S: 10; d_p_: <0.2 mm	Peel	0.72–3.52	0.84–5.72
		Seed	0.67–1.22	0.5–0.69
[112]	Solvent: ethanol:water (61:39%); t: 15 min; L/S: 0.05; amplitude: 85%; d_p_: <1 mm	Seeds	1.61	-
[37]	Solvent: methanol; t: 1 h; T: 25 °C; L/S: 10	Tomato	1.83–3.43	0.12–0.35
[36]	Solvent: methanol; t: 5 min; L/S: 1	Red-ripe peel	0.369 ^b^	-
		Green peel	0.299 ^b^	-
		Red-ripe seed	0.176 ^b^	-
		Green seed	0.133 ^b^	-
		Pomace	0.146 ^b^	-
[113]	Solvent: methanol; t: 1 h; T: 40 °C; L/S: 100; d_p_: <1 mm	Leaves	240	184
*Microwave-assisted extraction (MAE)*				
[111]	Solvent: water; L/S: 0.005; t: 60 min; power: 120 W	Pomace	17.87	
[114]	Solvent: methanol:water (50:50) t: 10 min; T: 90; L/S: 50; power: 0–500 W	Peel	78.06	65.85
[112]	Solvent: ethanol:water (63:37%); t: 15 min; T: 80 °C; L/S: 0.08; Frequency: 2.45 GHz; d_p_: <1 mm	Seeds	1.72	-
[38]	Solvent: ethanol:water (66.2:33.8) t: 2 min; T: 96.5 °C L/S: 50	Tomato	9.55	-
[25]	Solvent: ethanol (100%); t: 20 min; T: 180 °C; L/S: 45; d_p_: <0.85 mm	Rotten	66.8 ^b^	3.89 ^b^
[25]	Solvent: ethanol:water (47.4:52.6); t: 20 min; T: 180 °C; L/S: 22; d_p_: <0.85 mm	Rotten	43.9 ^b^	3.50 ^b^
*Accelerated solvent extraction*				
[16]	Solvent: water; t: 10 min; T: 45 °C; pressure: 600 MPa	Peels	49.92	-
*Enzymatic-assisted extraction*				
[50]	Solvent: ethyl acetate/enzyme; t: 1 h; T: 50 °C L/S: 5; enzyme: Celluclast:Viscozyme	Pomace	28.9 ^b^	-

^a^ Phenolic concentration in mg/g_fw_; ^b^ phenolic concentration in mg/g_dbe_; L/S—liquid-solid ratio in mL/g; d_p_—avarage particle diameter.

### 4.2. Recovering Energy through Anaerobic Digestion

Anaerobic digestion (AD) is a biological process in which microorganisms can break down biodegradable organic material in the absence of oxygen, producing biogas (CH_4_ and CO_2_) and remaining digestate (sludge with microorganisms and undegraded substrate and water). As can be seen in Table 1, tomato fruit residues (tomato rotten, green, fruit, and pomace) are mainly composed of organic matter (>80%). Thus, high biochemical methane potential (BMP) values are expected. Indeed, tomato residues have been studied for energy recovery. Some of the literature results are summarized in Table 6. BMP values range between 84.7–330, 124–369, and 28.8–365 mL_CH4_/g_VS_ for tomato pomace (peels and seeds), tomato fruit, and tomato plant (leaves and stems), respectively. The process complexity and the variations of the operational conditions are the main reasons for the large range of values reported. Indeed, the substrate-to-inoculum ratio (SIR) significantly affects the AD performance [62,129,130]. A SIR of 1, 2, 3, and 4 led to a methane yield of 56, 32, 48, and 29 mL_CH4_/g_VS_ for tomato pomace, respectively [129]. In contrast, lower SIR, such as 0.25 and 0.5, allowed it to reach a BMP value of 211 and 261 mL_CH4_/g_VS_, respectively [129]. High SIR causes an organic matter overload to the microbial population, and the anaerobic medium acidifies, inhibiting methanogenesis because methanogenic microorganisms are sensitive to pH. For SIR ratios higher than 1, acidic pH and VFA/TA (volatile fatty acid/total alkalinity) ratios in the unstable range (0.4–0.8) were reported at the final stage of the anaerobic digestion process [129]. So, the SIR ratio between 0.25 and 0.5 enhances the methane yield and also allows the maintenance of pH in the optimum range for organic matter degradation [62,129]. Moreover, higher SIR causes a delay in biogas production because more time is necessary to achieve enough methanogenic microorganisms in the inoculum that degrade the organic matter [62].

Lignocellulosic materials typically have a lower methane yield because lignin is difficult to biodegrade. Tomato plants (leaves and stems) are rich in lignin, as can be seen in Table 1; thus, lower BMP values are expected. However, Li et al. [146] reported a BMP value of 28.8 mL_CH4_/g_VS_, which is extremely lower when compared with other studies. These discrepant results are probably due to the SIR ratio (4) used by the authors.

Total volatile solids (tVS) concentration in the reaction mixture is also an important factor for AD. Camarena-Martínez et al. [62] evaluated the effect of tVS and stated that an increase from 13.0 g_VS_/L to 19.5 g_VS_/L leads to a methane production increase from 181 to 208 mL_CH4_/g_VS_. It is important to note that BMP rises as the tVS concentration increases until an optimal point; thus, an optimization is commonly required.

Anaerobic co-digestion (AcoD) is an alternative to single anaerobic digestion, allowing the management of multiple substrates simultaneously while producing biogas. Tomato residues have been tested with cattle manure, including cow manure, pig slurry [53,131,132,142,148] and corn stover [53,131,148], food waste [142], cucumber waste [29], and watermelon [131]. From these multiple combinations tested, a methane yield of about 300 mL/g_VS_ can be achieved [28,131,146]. Almeida et al. [5] performed a DoE to determine the best substrate combination for digesting rotten tomatoes, green tomatoes, and tomato plants. Only combinations with a high amount of tomato plants impaired the BMP results. Nonetheless, a maximum of 324 NmL_CH4_/g_VS_ was achieved with a mixture of 63% of rotten tomato, 20% of green tomato, and 17% of tomato plant [5]. Thus, a tomato industry can treat and valorize simultaneously the multiple residues generated.

Overall, tomato residues have shown a high potential for methane production through AD or AcoD. However, authors should invest more time in selecting and testing more combinations for AcoD, mostly thinking about the regional context and aiming to manage multiple regional residues, as proposed by Aravani et al. [131]

### 4.3. Closing the Loop through Composting Processes

Composting is a biological process in which microorganisms can break down biodegradable organic material in the presence of oxygen, releasing CO_2_, H_2_O, and heat, resulting in a stabilized material known as compost. Composting of tomato residue combined with dairy manure, wheat straw, and sewage sludge has been studied [61,63,80,81,149,150].

Tomato plant residues have been used as a bulk agent to maintain proper FAS in the composting mixture [63,81,149,150]. According to Kulcu [150], 60% of tomato stalks, 30% of separated dairy manure, and 10% of wheat straw are the optimum mixture ratio for composing, attaining suitable values of FAS (30%). The composting of two-phase olive-mill pomace was improved when higher contents of tomato stalks and poultry manure [149]. Şevik et al. [63] revealed high decomposition levels from composting mixtures composed of 5.0%, 67.1%, and 27.9% of olive pomace, dairy manure, and tomato stalks, respectively. The aerobic degradation of tomato stalks with sheep manure extended the thermophilic phase and enhanced the quality of compost regarding nutrients (P, K, N) and humic substances [80].

In another study, tomato green, rotten tomato, and tomato stalks were mixed with chicken manure (20%), and the composting of this mixture led to an organic matter loss of 36.4% [151]. The maturity and stability of the final compost were evaluated by CIELAB color space parameters (validated by physicochemical parameters) and it was concluded that the former method could be used to analyze the composting progress [151].

The combination of compost amendments (such as biochar, peat bog, and zeolite) with tomato stalks and chicken manure was studied [61]. Biochar was the best compost amendment due to its high surface area, improving the regulation of moisture content and free air space and, consequently, enhancing the environmental conditions for the growth of aerobic microorganisms.

Li et al. [152] evaluated the influence of the digestion time in an integrated anaerobic digestion and composting system. Briefly, tomato stalks and leaves were anaerobically degraded during 15, 30, and 45 days generating three different digestates. Those digestates were subjected to composting with corn stalks. The use of digestates formed within 30 and 45 days improved the germination index (a parameter used as a measure of compost maturation). Moreover, a reduction of 18.9-29.0% of greenhouse gas emissions was attained when digestates were composted. This study concluded that composting is an efficient and economical method to properly manage the digestate from AD of tomato residues.

## 5. Economic Analysis

**Figure 6:**
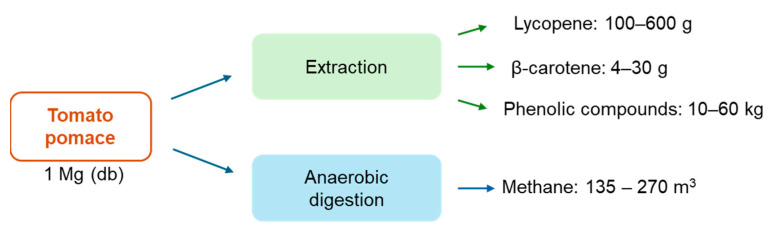
Range of recoverable products from 1 Mg of tomato pomace.

As mentioned before, many alternatives for managing and valorizing tomato residues have been studied. Multiple process yields have been reported according to the operating conditions. Nevertheless, Figure 6 summarizes the ranges of bioactive compounds and methane obtained through extraction and anaerobic digestion of tomato pomace. Indeed, the quantity of bioactive compounds corresponds to a small fraction of the tomato residues. However, the market of lycopene and β-carotene tends to increase with a compound annual growth rate (CAGR) of 5% and 4.3% from 2023 and 2030, respectively [153,154]. In addition, the demand for natural lycopene and β-carotene has increased due to the awareness of a healthy lifestyle. Although there is no established market value for natural extracts with bioactive compounds (lycopene and carotenoids), the commercialized lycopene and β-carotene present high market values. Thus, the studies with economic evaluation assumed high selling prices for extract rich in those compounds (30–50 USD/kg of extract) [73,98]. Overall, the high value of the compounds may compensate for the small amount recovered. A techno-economic analysis must be performed to evaluate the most profitable valorization strategy.

The profitability of each process and scenario proposed is commonly assessed by three main economic indicators: net present value (NPV), internal return rate (IRR), and payback time (PBT). NPV is a measure of the project profitability including the cash inflows and outflows associated with an investment at an established discount (interest) rate. IRR corresponds to the discount rate, which pays the investment cost at the end of the project (the discount rate at which NPV is zero). If the IRR is higher than the discount rate defined by the market, the project is economically viable. PBT represents the time needed to pay the project investment. Some authors have already evaluated the economic viability of applying supercritical fluid extraction for recovering extracts rich in lycopene and carotenoids from tomato and tomato pomace [98,155] and AcoD for obtaining biogas and digestate [53,156]. Different concentration of solids in the anaerobic reactor was also economically evaluated by some authors (wet if TS < 10% and dry if TS > 15%). Table 7 summarizes the economic data related to the valorization processes of tomato residues. As implied before, the economic viability of a project depends on many assumptions, including the lifetime and interest rate. The projects with extraction processes defined a lifetime of 10 years, while 20 years were specified for projects with anaerobic digestion processes. The interest rate ranged from 5.5 to 25% [98,155,157]. Except for the wet anaerobic co-digestion, all the processes were revealed to be economically viable. Thus, tomato residues can be managed using environmentally friendly technologies while producing profit through the recovery of natural and functional compounds (lycopene and carotenoids), energy (biogas), and digestate (used as fertilizer).

The extraction of lycopene and carotenoids leads to high revenues (111–10,300 kUSD/y) due to the high value of those compounds. Hatami and Ciftci [155] and Yadav et al. [157] reported very high prices for tomato extracts (423 USD/kg and 13,050 USD/kg, respectively), explaining the revenues reported (Table 7). A sensitivity analysis for the supercritical fluid extraction proposed by Hatami and Ciftci [155] revealed that extract price had the most significant impact on the NPV [158]. Nonetheless, NPV remains positive in the worst scenario [158].

Regarding AcoD, dry systems lead to profitable projects with NPV higher than 197 kUSD and IRR higher than the established discount rate (8%) [53,156]. Moreover, the conversion of methane in heat and electricity, including a combined heat and power (CHP) system, increased the NPV by 50% [156]. The revenues from the digestate accounted for about 50% of the total revenues [53,156]. Thus, all the products from anaerobic digestion have a high value. Indeed, digestate presents a high content of nutrients (N, P, and K) that can be used to replace a fraction of commercial fertilizers. The authors assumed prices for bio-methane, heat, electricity, and digestate of 0.54 USD/m^3^, 0.16 USD/kWh, 0.04 USD/kWh, and 30 USD/t, respectively [53,156]. A sensibility analysis revealed that the efficiency of the CHP, methane yield, and price of the products are the main variables affecting the NPV [53,156].

Only Scaglia et al. [73] evaluated the economic viability of a biorefinery for tomato pomace. SFE, followed by AD, allowed to obtain revenues higher than the manufacturing costs. Thus, applying biorefinery approaches may generate profits of 1450 kUSD/y [73]. Biorefineries with different feedstock also showed to be economically profitable [1]. This is a very good indicator for exploring biorefineries to manage and valorize tomato residues while the carbon loop (and nutrients) is closed. If the tomato processing industries adopt the valorization of tomato pomace, a positive contribution to the circular economy is expected by promoting the efficient use of resources, minimizing waste, and encouraging the recycling and reuse of by-products. By transforming waste into valuable secondary products, companies can lower disposal costs, reduce their environmental footprint, and create additional revenue. However, these processes might offer limited improvements in economic return in the short term due to initial investment costs. Long-term gains are expected as the industry adapts and scales these innovations, potentially leading to more substantial economic benefits and a more sustainable production model. Besides the economic analysis, in the literature, some recent studies have highlighted the need for a rigorous evaluation before using food residues, namely in terms of safety and risk assessment [159].

## 6. Conclusions and Future Perspectives

The tomato industry is a relevant socio-economic activity in the EU that generates diverse residues in large amounts. Tomatoes unfit for consumption (rotten and green), tomato plants, tomato seeds, tomato peels, and tomato pomace are among the most common residues widely studied, aiming at proper management and valorization. Extraction of β-carotene, lycopene, and phenolic compounds and anaerobic digestion for biogas production are the processes most widely studied in the literature. Although many studies have extracted carotenoids and phenolic compounds from tomato residues, there are many variables affecting the results hindering the comparison between different works. For this reason, comparative studies should be performed using the same residue, pretreatments, and analytical procedures to understand which extraction process maximizes the recovery of β-carotenes and phenolic compounds. Moreover, the results should be normalized by the initial biomass weight used as well as by the extract obtained to uniformize the units and allow the comparison between works. Both forms inform about the extraction performance, but in different relevant ways. The biorefinery approach has been gradually studied for tomato residues to maximize revenues and safeguard the environment. The integration of multiple processes, mainly to extract bioactive compounds, has been reported in the literature. Higher or similar efficiencies were reported by sequential processes when compared to stand-alone processes. Considering that good results were reported for extracting bioactive compounds, anaerobic digestion (or co-digestion), and composting, a more complex biorefinery, including those processes, must be investigated aiming to close the carbon (and nutrients) loop as suggested by the authors. The valorization of multiple residues simultaneously in co-digestion strategies using common agro-residues from a specific region should also be investigated, aiming to treat and valorize the waste streams in industrial plants. The techno-economic analysis shows good perspectives about recovering carotenoids from tomato residues because high profits were reported in the literature. However, more economic evaluations comparing different valorization processes and using the biorefinery approach should be performed to select the most profitable way of managing tomato residues. Even the drying effect on bioactive compound extraction should be economically assessed, assuring that higher efficiencies cover the drying operating costs. Before any recovery, safety assessment studies are necessary, particularly involving microbiological assessments or the determination of toxic contaminants in the residues. In addition, the life cycle assessment regarding tomato residue management is still lacking in the literature. However, these holistic data are of high relevance for understanding and selecting the best methods to valorize the waste streams. To sum up, more comparative studies, economic evaluations, and life cycle assessments of different strategies to valorize tomato residues will ensure a well-planned solution to reduce the cost of tomato industries with the management of these residues and boost the circular economy in the future.

## Figures and Tables

**Table 2:** Technology readiness level (TRL) of the main processes used for treating/valorizing tomato residues considering 3 different scales.

Refs.	Technology	Product	Technological Readiness		
			Lab Scale TRL1–3	Pilot Scale TRL4–6	Industrial Scale TRL7–9
[72]	Food processing	Animal feed			✓
[70]	Food processing	Tomato residues powder	✓		
[74]	Solid-state fermentation	Lycopene	✓		
[12,71]	Extraction	Lycopene	✓		
[12,71]	Extraction	β-carotene	✓		
[12,71]	Extraction	Phenolic compounds	✓		
[5,73]	Anaerobic digestion	Biogas	✓	✓	✓
[75]	Gasification	Syngas	✓		
[76]	Pyrolysis	Biochar	✓		
[77,78,79]	Pyrolysis	Bio-oil	✓		
[13]	Pyrolysis	Syngas	✓		
[80,81]	Composting	Compost	✓	✓	✓

**Table 6:** Biochemical methane potential (BMP—mL_CH4_/g_VS_) of tomato residues.

Ref.	Operational Conditions	Residue	BMP
[40]	SIR: 0.5; t: 40 d	Pomace	218 ^a^
[44]	SIR: 0.4; tVS: 49.4 g_VS_/L; Inc: Y; t: 30 d	Pomace	330 ^a^
[87]	WV: 40%; t: 90 d	Pomace	108
[129]	WV: 0.8 L; SIR: 0.5; tVS: 60 g_VS_/L	Pomace	261
[131]	SIR: 0.25–1; T: 37 °C; t: 39–45 d	Pomace	305
[132]	WV: 60%; SIR: 0.5; t: 48 d	Pomace	151
		Plant	99.4
[133]	WV: 50%; SIR: 1; tVS: 14.4 g_VS_/L; t: 22 d	Pomace	250
[134]	WV: 75%; t: 40 d	Pomace	84.7
[27]	WV: 75%	Tomato	341
[29]	tVS: 1.25 g_VS_/L; Nu: Y; t: ~72 d	Tomato	299
[55]	WV: 75%; SIR: 2; T: 36 °C; Nu: Y	Tomato	124.4
[135]	-	Tomato	231
[136]	Hohenheim Batch Test method	Tomato	370 ^a^
[137]	Nu: Y; t: 100 d	Tomato	211
[138]	WV: 75%; SIR: 0.5	Tomato	295 ^a^
[139]	-	Tomato	369
[12]	WV: 20%; SIR: 0.5; tVS: 34.5 g_VS_/L; t: 30 d	Rotten tomato	285 ^a^
		Green tomato	232 ^a^
		Plant	141 ^a^
[140]	WV: 20%; SIR: 0.5; tVS: 34.5 g_VS_/LAddition of biomass fly ash	Rotten tomato	261 ^a^
[55]	WV: 70%; SIR: 4;	Plant	124
[62]	WV: 67%; SIR: 0.5; tVS: 19.5 g_VS_/L; Nu: Y; t: ~30 d	Plant	214
[141]	SIR: 0.48; T: 40 °C	Plant	211 ^a^
[142]	SIR: 0.3; T: 52 °C; t: 7 d	Plant	184
[143]	SIR: 0.5; WV: 62.5% T: 37 °C; t: 28 d	Plant	365
[144]	t: 60 d	Plant	97–125 ^a^
[145]	WV: 75%	Plant	320
[146]	SIR: 4	Plant	28.8
[147]	tVS: 10 g_VS_/L; Nu: Y	Plant	242

^a^ NmL_CH4_/g; WV—working volume; SIR—substrate-to-inoculum ratio; tVS—total volatile solid concentration in the reaction mixture (substrate + inoculum); Nu—nutrients; Inc—incubation; Y—indicate the presence of nutrients or incubation; t—time in days (d).

**Table 7:** Capital and manufacturing costs, revenues, and economic indicators of different valorization processes for tomato residues.

Ref.	Residues	Process	Product(s)	Processing Capacity	Capital Cost	Manufacturing Cost	Revenues	NPV	IRR	PBT
					kUSD	kUSD/y	kUSD/y	kUSD	%	y
[98]	Tomato	SFE	Lycopene Oil with carotenoids	100 kg/batch		28.8	176			
		SFE	Oleoresin	100 kg/batch		26.4	111			
		Soxhlet extraction	Oleoresin	100 kg/batch		79.3	215			
[155]	Pomace	SFE	Lycopene	100 L extractor	1770	1800	10,300	41,800		0.32
[157]	Tomato	Extraction using surfactant	Lycopene	470 kg/d	28.3	44.5	60.4	9.5		3.7
[53]	Tomato residues + dairy manure + corn stover	Wet AcoD	Electricity Digestate	3.9 m^3^/d	192	11.7	35	−26		
		Dry AcoD	Electricity Digestate	3.9 m^3^/d	181	10.4	65.9	264	11.7	10.9
[156]	Tomato residues + dairy manure + corn stover	Dry AcoD	Biogas Digestate	8000 Mg/y	523	70	183	401	20	
[156]		Dry AcoD	Electricity Heat Digestate	8000 Mg/y	603	70	215	600	23	
[73]	Pomace	SFE AD	Lycopene Carotenoids Digestate Biogas	396 Mg/y	2000	1100	2550			

NPV—net present value; IRR—internal return rate; PBT—payback time.

## Data Availability

No new data were created or analyzed in this study. Data sharing is not applicable to this article.
